# Effects of dietary nitrate supplementation on microvascular physiology at 4559 m altitude – A randomised controlled trial (Xtreme Alps)

**DOI:** 10.1016/j.niox.2019.10.004

**Published:** 2020-01-01

**Authors:** Andrew F. Cumpstey, Philip J. Hennis, Edward T. Gilbert-Kawai, Bernadette O. Fernandez, Daniel Grant, William Jenner, Matthieu Poudevigne, Helen Moyses, Denny ZH. Levett, Alexandra Cobb, Paula Meale, Kay Mitchell, Helmut Pöhnl, Monty G. Mythen, Michael PW. Grocott, Daniel S. Martin, Martin Feelisch

**Affiliations:** a Critical Care Research Area, Southampton, NIHR Southampton Biomedical Research Centre, Southampton General Hospital, Tremona Road, Southampton, SO16 6YD, UK; b Anaesthesia and Critical Care Research Unit, University Hospital Southampton NHS Foundation Trust, Tremona Road, Southampton, SO16 6YD, UK; c Integrative Physiology and Critical Illness Group, Clinical and Experimental Sciences, University of Southampton, Tremona Road, Southampton, SO16 6YD, UK; d UCL Centre for Altitude, Space and Extreme Environment (CASE) Medicine, UCLH NIHR Biomedical Research Centre, Institute of Sport Exercise & Health, 170 Tottenham Court Road, London, W1T 7HA, UK; e Clinical & Experimental Sciences, Faculty of Medicine, NIHR Southampton Biomedical Research Centre, University of Southampton and University Hospital Southampton NHS Foundation Trust, Tremona Road, Southampton, SO16 6YD, UK; f Warwick Medical School, Division of Metabolic and Vascular Health, University of Warwick, Gibbet Hill Road, Coventry, CV4 7AL, UK; g AURAPA, Paul-Heidelbauer-Straße 26, 74321, Bietigheim-Bissingen, Germany

**Keywords:** Microcirculation, Nitrate, Nitrite, Altitude, Nitric oxide, Hypoxia

## Abstract

Native highlanders (e.g. Sherpa) demonstrate remarkable hypoxic tolerance, possibly secondary to higher levels of circulating nitric oxide (NO) and increased microcirculatory blood flow. As part of the Xtreme Alps study (a randomised placebo-controlled trial of dietary nitrate supplementation under field conditions of hypobaric hypoxia), we investigated whether dietary supplementation with nitrate could improve NO availability and microvascular blood flow in lowlanders. Plasma measurements of nitrate, nitrite and nitroso species were performed together with measurements of sublingual (sidestream dark-field camera) and forearm blood flow (venous occlusion plethysmography) in 28 healthy adult volunteers resident at 4559 m for 1 week; half receiving a beetroot-based high-nitrate supplement and half receiving an identically-tasting low nitrate ‘placebo’. Dietary supplementation increased plasma nitrate concentrations 4-fold compared to the placebo group, both at sea level (SL; 19.2 vs 76.9 μM) and at day 5 (D5) of high altitude (22.9 vs 84.3 μM, p < 0.001). Dietary nitrate supplementation also significantly increased both plasma nitrite (0.78 vs. 0.86 μM SL, 0.31 vs. 0.41 μM D5, p = 0.03) and total nitroso product (11.3 vs. 19.7 nM SL, 9.7 vs. 12.3 nM D5, p < 0.001) levels both at sea level and at 4559 m. However, plasma nitrite concentrations were more than 50% lower at 4559 m compared to sea level in both treatment groups. Despite these significant changes, dietary nitrate supplementation had no effect on any measured read-outs of sublingual or forearm blood flow, even when environmental hypoxia was experimentally reversed using supplemental oxygen. In conclusion, dietary nitrate supplementation does not improve microcirculatory function at 4559 m.

## Highlights

• Xtreme Alps is a randomised controlled field study of dietary nitrate at altitude.
• Dietary nitrate significantly increased plasma nitrate, nitrite & nitroso species.
• No changes in sublingual blood flow were seen in response to high dietary nitrate.
• Dietary nitrate did not alter forearm blood flow under any experimental condition.
• Dietary nitrate supplementation did not improve microcirculatory function at 4559 m

## 1 Introduction

Atmospheric pressure, and consequently the partial pressure of oxygen, both decrease with increasing altitude. This hypobaric hypoxia can cause many challenges to those visiting or living in highland regions, including serious illnesses which can prove fatal if left untreated [1,2]. Yet given appropriate time to acclimatise (the process by which an individual adapts physiologically to environmental changes such as hypobaric hypoxia), humans have been able to successfully reach extreme altitudes despite remarkably low environmental levels of oxygen [3]. Intuitively, the factors considered most important to mounting a successful acclimatisation response are usually those that result in global circulatory changes and enhance tissue oxygen delivery; e.g. increased heart rate, cardiac output and erythropoiesis [2]. However, detailed physiological profiling of a number of world-class high altitude mountaineers showed remarkably little differences in all reported measures of global circulatory performance [4]. Similarly, Sherpas, who are renowned for their exceptional performance at extreme altitudes, display only modest levels of erythropoiesis [[5], [6], [7], [8]]. However, both elite high altitude mountaineers and Sherpas demonstrate significantly higher muscle capillary densities than lowlander controls, suggesting that excellent altitude performance might, at least in part, be due to differences in microcirculatory function (such as exercise-induced angiogenesis or simply smaller fiber atrophy) [4,9]. The proportion of capillaries actually supporting red blood cell flux may be even more important than muscle oxygen diffusing capacity in determining overall microvascular oxygen pressures [10].

The microcirculation refers to the smallest blood vessels in the body, typically those less than 100 μm in diameter, and microcirculatory dysfunction is increasingly being implicated in the development of critical illnesses, sepsis and other shock states [11,12]. Novel non-invasive techniques, such as sidestream dark-field (SDF) imaging, have only recently allowed direct visualisation of the human microcirculation [13,14]; particularly, the sublingual microcirculation, which is easily monitored and might offer a convenient proxy for other vascular beds. For example, the proportion of vessels flowing in the sublingual bed closely coheres with the latest measurements within skeletal muscle (approximately 80%) [15]. Recent studies using these techniques suggested that microcirculatory blood flow significantly decreased in lowlanders at altitude, particularly in small (<25 μm) diameter vessels [16,17]. However at 5300 m, Sherpas demonstrated significantly higher microcirculatory blood flow than resident lowlanders [18]. Similarly, forearm blood flow (an indirect measure of microcirculatory status at the level of resistance arteries) in Sherpas was more than twice that in lowlanders, and these responses were closely associated with significant increases in circulating concentrations of plasma nitrate and nitrite, indicative of increased nitric oxide (NO) formation and/or bioavailability [19].

NO is a ubiquitous cellular messenger and effector molecule (belonging to a group of small molecules often referred to as ‘gasotransmitters’) with potent vasoactive and anti-inflammatory properties [[20], [21], [22]]. Physiologically, NO is produced from l-arginine by a family of isoenzymes known as ‘nitric oxide synthases’ (NOS) (usually co-expressed with arginase enzymes in smooth muscle) in an oxygen-dependent process; an alternative pathway is thought to proceed through serial reduction of inorganic nitrate (NO_3_^−^) to nitrite (NO_2_^−^) and then finally to NO. This latter process is promoted by hypoxic conditions because oxygen normally inhibits the reduction process [23], which might explain the significant increases in NO metabolism observed at altitude [24]. Dietary nitrate may be converted to NO through a similar mechanism; after being reduced to nitrite by oral commensal bacteria, it may be further reduced to NO by chemical processes inside the stomach and enzymatic bioactivation in the vasculature and other tissues [[25], [26], [27]]. Inorganic nitrate supplementation with beetroot juice has already been shown to improve vascular control and elevate skeletal muscle oxygen delivery (particularly in fast-twitch II fibres) in exercising rats [28].

The Xtreme Alps expedition was a double-blinded randomised placebo-controlled trial to investigate whether dietary nitrate supplementation could improve performance at altitude, and a full study methodology has been published elsewhere [29]. The first results from the Xtreme Alps study have demonstrated that this dietary supplementation is safe and successfully increases pulmonary NO availability; but surprisingly without significantly affecting measures of global circulatory function, such as heart rate or blood pressure [30]. In this second report, we hypothesised that if dietary nitrate supplementation could increase circulating NO levels as well as increasing pulmonary NO availability, then it might perhaps improve microcirculatory function at altitude.

## 2 Methods

### 2.1 Study settings and participants

Ethical approval for the Xtreme Alps study was obtained from Research Ethics Committees at both University College London, UK and the University of Turin, Italy, which manages a research laboratory inside the Capanna Regina Margherita (‘Margherita Hut’) on the summit of Monte Rosa (altitude 4559 m) where the study was performed in August 2010. Complete methods of the Xtreme Alps expedition and protocols have been described elsewhere [29]. Healthy volunteers (aged 18 years and above) were recruited primarily from an electronic mailing list of individuals interested in altitude research and physiology. Twenty-eight volunteers participated in total (75% male); with a mean age of 28.9 years (range 21–40), mean weight 73.3 Kg (±11.6), mean height 1.76 m (±0.08), and mean BMI 23.6 (±2.69); 3.6% were smokers and 75% had previously ascended above 3000 m. All volunteers gave written informed consent and completed a health screening process (as detailed in Ref. [29]) before participating; anyone considered unfit to perform exercise at high altitude (as previously explained [31]) was excluded.

**Fig. 1:**
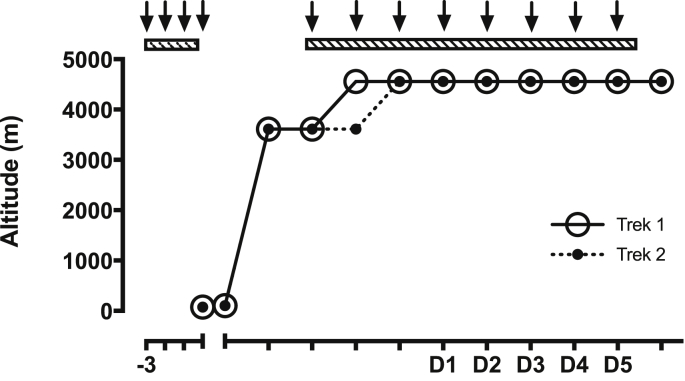
Ascent profile used during the Xtreme Alps expedition as shown previously in Ref. [30]. Trek 1 and Trek 2 are represented by solid and dotted lines respectively. Trek 1 ascended from the Gnifetti Hut (3611 m) to the Margherita Hut (4559 m) one day earlier than Trek 2 due to inclement weather conditions. Each arrow represents the administration of 3 doses of either treatment or placebo supplement (1 dose before every meal each day), starting 3 days prior to each testing period. SL = sea level testing, D1 – D5 = altitude testing days 1–5.

Baseline measurements were taken in London (altitude 75 m, mean barometric pressure 100.5 kPa, mean laboratory temperature 24.1 °C and mean oxygen partial pressure 19.7 kPa). Participants were separated into 2 trekking groups (Trek 1 and Trek 2) based on their availability, with Trek 1 starting their ascent seven days before Trek 2. All trekkers initially flew into Milan (102 m) and remained there overnight before starting to ascend. The following day, participants travelled by road to Alagna (1205 m), ascended to Punta Indren (3250 m) using lifts and then walked to the Gnifetti Hut (3611 m). We planned for both trekking groups to acclimatise at 3611 m for 3 nights before continuing to ascend on foot to the Margherita Hut (4559 m). However, incoming storms forced Trek 1 to ascend to 4559 m one day earlier than Trek 2, i.e. after spending only two nights at 3611 m. After reaching the Margherita Hut, participants remained at 4559 m for the duration of the study (eight nights for Trek 1 & seven for Trek 2, see Fig. 1) before descending. In the laboratory at 4559 m the mean barometric pressure was 78.1 kPa, mean ambient temperature 22.6 °C and mean partial pressure of oxygen calculated to be 15.1 kPa.

### 2.2 Intervention and randomisation

Participants were allocated at random (in accordance with the CONSORT 2010 guidelines, http://www.consort-statement.org/) to receive either an intervention or placebo version of a custom formulated all-natural beetroot/fruit juice drink (produced and provided by Aurapa GmbH, Bietigheim-Bissingen, Germany) starting 72 h before and continuing for the duration of each testing period (i.e. both sea level and altitude). The low nitrate supplement (placebo group) contained only 11.5 μmol/kg per day (split into three equal doses to be taken at each mealtime) as the majority of the nitrate content of the beetroot portion had been removed by a microbial denitrification process; whilst in the high nitrate supplement (intervention/treatment) group the total daily nitrate content was approximately 0.10–0.18 mmol/kg/day (again split over three doses for consumption together with meals) [30]. This intervention dose is very similar to levels demonstrated previously to improve exercise performance at sea level [32,33]. Participants and investigators both remained blinded to group allocations for the duration of the study, including the analysis of the primary data sets. All other meals during the study period were standardised to minimise extra nitrate intake in both groups, and the nitrate content of each meal was measured and recorded as has been reported previously [30].

### 2.3 Collection of blood samples and measurement of hemoglobin

Fasted venous blood samples (5 ml) were taken at sea level and on the 1st, 3rd and 5th testing mornings (D1, D3 & D5) during the study at altitude, using EDTA-containing BD Vacutainer™ tubes. Each of these samples was immediately centrifuged at 800×*g* for 15 min, and 1 ml of plasma and the red blood cell pellet were both aliquoted into individual cryovials and frozen at −40 °C for transport back to sea level where they were stored at −80 °C until later analysis.

Blood haemoglobin (Hb) concentrations were quantified in the field immediately after sampling using a handheld photometric device (HemoCue® Whole Blood Haemoglobin System, HemoCue AB, Angelhoim, Sweden). Measurement ‘cuvettes’ for the HemoCue were always stored within a thermostatically controlled storage unit in accordance with the manufacturer's recommendations.

### 2.4 Plasma biomarker analysis

All plasma biomarker concentrations were quantified after reaction with an excess of N-ethylmaleimide (NEM, in PBS; 10 mM final concentration) immediately after frozen plasma aliquots were thawed. To quantify plasma nitrite and nitrate concentrations, NEM-treated samples were deproteinized with methanol (1:1) and centrifuged at 16,100×*g* for 10 min before undergoing analysis by high-pressure liquid chromatography (HPLC) using a dedicated nitrite/nitrate analyzer (ENO20, Eicom). All sample analysis was performed with repeated daily calibrations and staggered to ensure processing times were similar, and all reported values were corrected for background levels of nitrite/nitrate. Total nitroso product concentrations were quantified by group-specific denitrosation of NEM-treated EDTA plasma samples after injecting samples incubated with acidic sulfanilamide directly into an acidic triiodide-containing reaction chamber and measuring the NO produced (following reduction of protein nitroso-species) by gas phase chemiluminescence (CLD 77am sp, EcoPhysics) as described previously [34]. Circulating plasma levels of l-arginine were measured spectrophotometrically (SpectraMax M5, Molecular Devices, Sunnyvale, CA, USA) using a commercial assay (L-Arg ELISA, CEB938Ge 96, Cloud-Clone Corp, Wuhan, China). Plasma arginase activity was also measured spectrophotometrically (Sigma-Aldrich MAK112, Dorset, UK).

### 2.5 Sublingual microcirculatory function

The sublingual microcirculation was imaged whilst resting in a supine position by sidestream dark field (SDF) imaging; a non-invasive hand-held video microscope (MicroVision Medical, Amsterdam, Netherlands) was placed under participants' tongues with the camera-tip pointing sublingually. The probe was moved around gently until clear images of the microcirculation were displayed, then retracted as much as possible without losing these images to minimise pressure-flow artefacts. Four repeat 20-s films were recorded from each subject, first at sea level and then again after residing at 4559 m for at least 4 days. These were assessed later using the Automated Vascular Analysis (AVA) 3.0 microcirculatory analysis software (MicroVision Medical, Amsterdam, Netherlands) by two trained and experienced investigators who were both blinded to the subjects’ identity and also to the location of each recording (either sea level or altitude).

Microcirculatory Flow Index (MFI) scores were calculated by vessel size in accordance with recognized guidelines [35]. Initially, each video image was divided into quadrants and all visualized blood vessels were sized into small (<25 μm), medium (26–50 μm) or large blood vessels (51–100 μm) using a calibrated microgrid. An overall MFI score was calculated for each vessel size using an internationally recognized flow grading system (0 for no flow, 1 for intermittent flow, 2 for slow flow or 3 for continuous/normal flow), first averaging the 4 quadrant scores in each individual recording and then averaging the overall scores obtained from each recording, either at sea level or at altitude.

The AVA 3.0 software was also used to calculate the vessel density. The position of blood vessel walls needs to be deduced in SDF imaging using changes in contrast between hemoglobin near the very edge of blood vessels and surrounding tissues. After image stabilization, time averaging of individual film frames filled interruptions in perceived vessel walls caused by plasma gaps and white blood cells. This slope of change in color was then used to define and trace out blood vessel edges automatically, allowing the software to calculate small vessel density (SVD; <25 μm diameter), medium vessel density (MVD; >25 μm diameter) and total vessel density (TVD; TVD = SVD + MVD), measured in mm ^**.**^ mm^−2^.

### 2.6 Forearm blood flow measurements

All participants underwent a non-invasive assessment of forearm blood flow in their dominant arm using strain gauge venous occlusion plethysmography (EC6 with rapid cuff inflator EC20/AG101 and NIVP3 software; D. E. Hokanson, Inc., Bellevue, WA, USA). An appropriately sized mercury-in-silastic strain gauge was placed just distal to the antecubital fossa, where the forearm circumference was largest, as the subject sat with their arm resting on styrofoam blocks elevated to the level of the right atrium. Initially, blood pressure cuffs placed around the wrist (inflated to 250 mmHg) and the upper arm (inflated to 50 mmHg) were used to exclude the hand circulation and occlude venous outflow from the forearm, and blood flow measurements as well as oxygen saturation levels were recorded at rest. Both cuffs were then deflated and after resting for 1 min, participants completed a standard forearm exercise protocol (repeated hand-squeezes of a foam ball at a frequency of 0.5 Hz for 2 min). Immediately following this handgrip exercise, both cuffs were re-inflated and subsequent circumference changes recorded. With venous outflow occluded, continuous arterial inflow led to linear increases in forearm circumferences, which are reported as the percentage changes of the slopes per minute generated. In accordance with classical venous occlusion plethysmography, five repeat values were recorded for each measurement. However, as described by our group previously [36], although repeated measures protocols such as this depict reliability and reproducibility of the technique being employed when recording baseline levels, averaging in this way actually provides little information about our primary interest, microcirculatory vessel recruitment and the resultant increases in blood flow, after an exercise intervention aimed at stimulating metabolic demand and thus maximising regional blood flow. The reason for this is that the flow increases observed are transient in most individuals. Consequently, here we only report and analyse initial slope increases, which are indicative of the Maximal Flow Response [36].

After taking measurements under ‘normal’ environmental conditions (i.e. 21% oxygen at sea level, and environmental hypobaric hypoxia at 4559 m altitude), all participants underwent further testing with these environmental conditions experimentally ‘reversed’. Specifically, after normal sea level measurements were taken, all participants were subjected to breathing a hypoxic gas mixture (12% oxygen) for 45 min before repeating the measurement protocol; and similarly, measurements were repeated at 4559 m after participants breathed 35% oxygen for 45 min. All responses were normalised to total forearm volume, determined individually by volume displacement.

### 2.7 Analysis plan

Statistical analysis was performed using linear mixed modelling in STATA 11 (http://www.stata.com) to account for the multiple time points at which measurements were taken at altitude and the different ascent profiles of each trek group. All p values report the treatment effect in response to taking the high nitrate dietary supplement unless otherwise specified separately in the text. All reported variables were either normally distributed, or converted into normally distributed data after logarithmic transformation (where transformation was performed this is specified in Table 2). Significance was assumed when p < 0.05, and a sensitivity analysis was performed with any missing values replaced by mean values to verify the model. Where variables were not normally distributed before transformation, descriptive values are reported as median (interquartile range) as appropriate to keep values in the original units for ease of understanding of the reader. Values for all variables that were normally distributed are reported as mean (±standard deviation).

**Table 1:** Descriptive table for sea level (SL) and respective testing days at altitude. Haemoglobin samples were taking during the first 3 days at 4559 m, plasma samples were taken on 1st (D1), 3rd (D3) and 5th (D5) testing days at 4559 m. Data are presented as median (IQR) or mean (±SD) as appropriate. RxNO – total nitroso products; cGMP – cyclic guanosine monophosphate.

		Placebo	Treatment
Haemoglobin (g/dL)	SL	15.0 (±1.1)	15.0 (±0.9)
	MH	16.1 (±1.4)	15.8 (±1.0)
Plasma Nitrate (μM)	SL	19.2 (16.0–22.3)	76.9 (68.3–110.6)
	D1	20.9 (17.3–22.8)	85.7 (70.5–102.0)
	D3	19.9 (16.9–22.9)	83.0 (74.2–95.2)
	D5	22.9 (22.0–26.4)	84.3 (66.9–101.0)
Plasma Nitrite (μM)	SL	0.779 (0.682–0.863)	0.856 (0.623–1.594)
	D1	0.332 (0.205–0.387)	0.355 (0.303–0.451)
	D3	0.311 (0.228–0.387)	0.378 (0.301–0.448)
	D5	0.312 (0.270–0.343)	0.409 (0.354–0.447)
Plasma RxNO (nM)	SL	11.3 (8.6–13.6)	19.7 (12.5–29.1)
	D1	10.8 (9.6–11.5)	15.0 (12.8–15.9)
	D3	10.6 (7.9–12.7)	11.9 (11.3–17.5)
	D5	9.7 (9.1–11.0)	12.3 (11.7–16.6)
Plasma cGMP (nM)	SL	234.8 (±70.8)	207.5 (±67.4)
	D1	144.6 (±45.4)	174.4 (±73.7)
	D3	161.2 (±54.6)	192.0 (±99.1)
	D5	203.0 (±78.1)	164.3 (±61.3)
Plasma l-Arginine (μg/ml)	SL	6.3 (4.7 - 8.7)	8.4 (2.6 - 14.8)
	D3	7.5 (5.7 - 16.9)	9.4 (5.8 - 33.0)
	D5	8.9 (4.5 - 15.7)	7.4 (6.1–36.7)
Plasma Arginase activity (Units/L)	SL	12.8 (±3.5)	14.9 (±3.6)
	D3	12.4 (±2.4)	12.5 (±2.7)
	D5	12.1 (±1.7)	12.7 (±2.4)
Plasma Protein concentration (g/L)	SL	79.5 (±15.4)	91.6 (±15.3)
	D1	89.4 (±30.1)	92.4 (±20.2)
	D3	84.4 (±24.4)	85.6 (±28.6)
	D5	79.8 (±23.5)	77.2 (±23.2)

**Table 2:** Regression coefficients for each plasma biomarker showing: regression coefficient, 95% confidence interval (95% CI) and p-values for the effect the treatment (dietary nitrate supplementation) had on each measured variable. The treatment (dietary nitrate supplementation) group displayed significant higher levels of plasma nitrate, nitrite, RxNO and arginase activity.

Variable	Regression coefficient (95% CI)	p-value
Haemoglobin	0.02 (−0.77, 0.82)	0.95
Log Plasma Nitrate	1.5 (1.3, 1.7)	<0.001
Log Plasma Nitrite	0.3 (0.04, 0.6)	0.03
Log Plasma RxNO	0.5 (0.2, 0.8)	<0.001
Plasma cGMP	−1.2 (−28.0, 25.6)	0.93
Log Plasma l-Arginine	0.11 (−0.51, 0.72)	0.73
Plasma Arginase	2.1 (0.11, 40)	0.04
Plasma Protein	0.77 (−10.7, 12.2)	0.90

## 3 Results

As reported previously, 1 participant was evacuated soon after arriving at 4559 m for medical reasons and did not complete any testing at altitude [30]. All other participants were able to undergo testing at altitude and all experimental protocols were conducted successfully.

### 3.1 Hemoglobin and plasma markers

Mean hemoglobin concentration slightly increased with altitude exposure in both groups, and this effect was not influenced by the nitrate intervention (see Table 1).

Dietary nitrate supplementation increased plasma nitrate concentrations 4-fold compared to the placebo group, both at sea level and at altitude (p < 0.001). Nitrate supplementation also significantly increased both plasma nitrite (p = 0.03) and total nitroso product (p < 0.001) concentrations, both at sea level and at altitude. However, fasting plasma nitrite concentrations were more than 50% lower at 4559 m compared to sea level in both the treatment and the placebo groups (see Table 1, Table 2).

As expected, cGMP concentrations also decreased on arrival at altitude in both groups, but there was no significant difference in cGMP levels with high nitrate treatment (see Table 1, Table 2). Similarly, dietary nitrate supplementation did not significantly alter plasma l-arginine or plasma protein concentrations either (p = 0.73 and 0.90 respectively), but mean plasma arginase activity was significantly higher (p = 0.04) in the high nitrate treatment group (see Table 1, Table 2).

### 3.2 Sublingual blood vessel density and blood flow

**Fig. 2:**
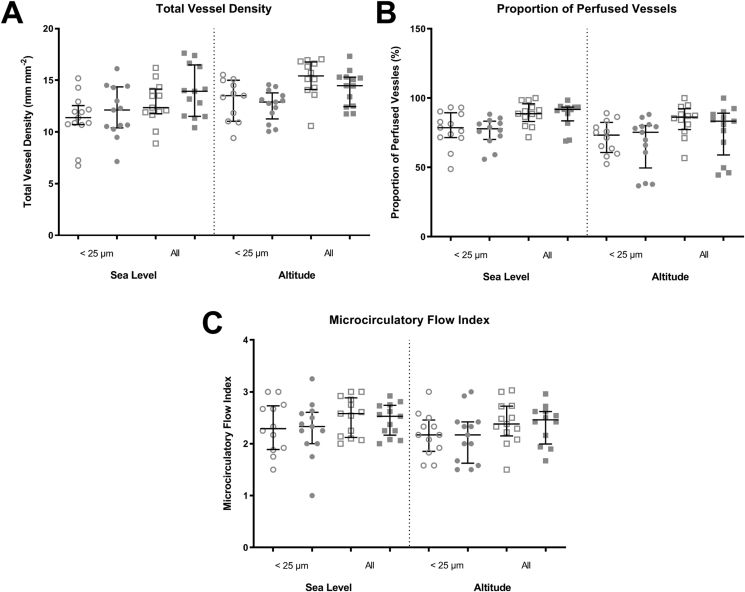
Comparison of microcirculatory function at sea level and high altitude. A = Total Vessel Density (TVD) for vessels that are < 25 μm diameter or all vessels, at sea level and at 4559 m altitude. B = Proportion of Perfused Vessels (PPV) for vessels that are < 25 μm diameter or all vessels, at sea level and at 4559 m altitude. C = Microcirculatory Flow Index (MFI) for vessels that are < 25 μm diameter or all vessels, both at sea level at 4559 m altitude. Open points represent the placebo (low nitrate dietary supplement) group; solid boxes represent the treatment (high nitrate dietary supplement) group. Circular points represent vessels <25 μm in diameter, square points represent all vessels.

The Total Vessel Density (TVD), Proportion of Perfused Vessels (PPV) and Microcirculatory Flow Index (MFI) for all measured vessels, and specifically for the smallest measured blood vessels (defined as those < 25 μm in diameter), both at sea level and at altitude, are shown in Fig. 2. Corresponding regression and significance data is detailed in Table 3.

**Table 3:** Regression coefficients for sublingual microcirculatory data measured with sidestream dark field (SDF) imaging; regression coefficient, 95% confidence interval (95% CI) and p values for the effect of treatment with high nitrate dietary supplementation on Total Vessel Density (TVD), Proportion of Perfused Vessels (PPV) and Microcirculatory Flow Index (MFI) are given below. There was no significant change seen in any measurement of sublingual microcirculatory flow in response to treatment with high nitrate dietary supplementation.

Variable (vessel size)	Regression coefficient (95% CI)	p-value
TVD (Small)	0.55 (−1.9, 3.0)	0.66
TVD (All)	1.12 (−0.37, 2.62)	0.14
PPV (Small)	−7.23 (−19.1, 5.3)	0.26
PPV (All)	−6.25 (−18.3, 5.8)	0.31
MFI (Small)	−0.05 (−0.3, 0.2)	0.7
MFI (All)	−0.02 (−.02, 0.2)	0.8

Despite the significant biochemical increases seen in response to high nitrate dietary supplementation described above, no significant differences were seen in any measure of sublingual microcirculatory function in response to treatment with high nitrate supplementation.

### 3.3 Forearm blood flow measurements

**Fig. 3:**
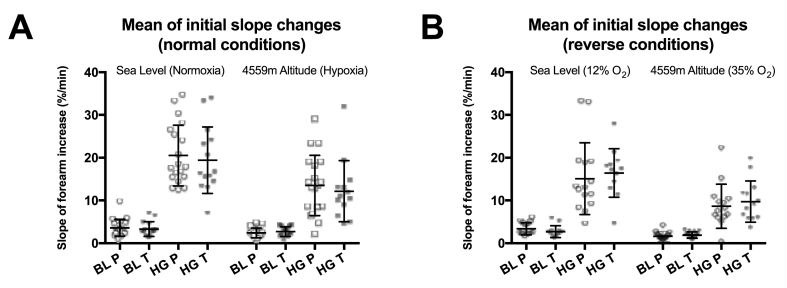
Forearm blood flow data from sea level and altitude. Each graph shows the increase in the initial forearm circumference caused by the inflow of arterial blood into newly opened microcirculatory vessels before (Baseline - BL) and after (Handgrip - HG) 2 min of standardised exercise both at Sea Level and at 4559 m Altitude. Overlying error bars represent the mean ± SD. Subjects taking the placebo (P) and treatment (T) supplements are represented by open and solid data points, respectively. Graph A = normal environmental conditions; i.e. normoxia at sea level represented by circular points, and hypoxia at altitude represented by squares. Graph B = reverse environmental conditions; i.e. after breathing 12% oxygen for 45 min at sea level (to mimic acute altitude exposure) represented by hexagonal data points, and breathing 35% oxygen for 45 min at 4559 m (to mimic being in London) represented by diamonds.

The handgrip protocol successfully resulted in measureable increases in forearm blood flow after a standardised exercise protocol, both at sea level and at altitude. At sea level, large increases in blood flow were observed; from 3.3 (±1.5) %/min to 19.9 (±7.0) %/min in the placebo group and from 3.3 (±1.8) %/min to 19.1 (±8.0) %/min in the treatment group. Similar changes were also seen at 4559 m, although both the placebo and the treatment groups displayed more moderate increases in peak forearm blood flow with exercise at altitude (see Fig. 3).

As with all reported measures of sublingual microcirculatory function above, the increase in forearm microcirculatory blood flow with exercise did not change with dietary nitrate supplementation in any measureable way, neither at sea level nor at altitude. Similarly, when environmental conditions were experimentally reversed by breathing 12% oxygen for 45 min at sea level or by inhaling 35% for 45 min at 4559 m altitude, there was still no significant difference in forearm blood flow between the placebo and treatment groups (see Fig. 3). Furthermore, the regression data from our statistical model revealed that the dietary nitrate intervention had no significant effect on forearm blood flow under any of the experimental conditions tested (data not shown).

## 4 Discussion

Our findings show that dietary nitrate supplementation significantly increases circulating levels of nitrate, nitrite and nitroso product concentrations, both at sea level and at 4559 m altitude. However, despite these significant biochemical changes, there was no demonstrable improvement in any measure of sublingual microcirculatory function or forearm blood flow (either at rest or after handgrip exercise, and under normal or reversed environmental conditions), suggesting that overall NO bioavailability remained unaltered both at sea level and at altitude. In a number of previous studies, dietary nitrate supplementation has been demonstrated to increase plasma nitrite (and/or nitrate) concentrations and result in improved running performance, walking performance, and cycling performance under conditions of normobaric hypoxia [[37], [38], [39], [40]]. However, almost all of these studies investigated the effect of dietary supplementation on the function of relatively large muscle groups or even whole body performance. Few studies have investigated the effects of nitrate supplementation on the microcirculation, even though microcirculatory adaptations may be crucial to how Sherpas can perform so well under extreme hypoxia [18].

A similar study to ours has recently also reported no significant change to forearm blood flow during acute exposure to a simulated altitude of 4300 m, despite increasing plasma nitrate levels 10-fold [41]. Interestingly, the single dose of 15 mmol nitrate used in that trial did not change peripheral oxygen saturations (SpO_2_) either despite increasing exhaled NO levels (PE_NO_) by 24%, also supporting previous findings of the Xtreme Alps study [30]. However, unlike the Xtreme Alps study, that trial used a single dose of nitrate and a relatively short period of simulated hypobaric hypoxia, which is likely to be a very different physiological stimulus to chronic field exposure to hypobaric hypoxia as in the Xtreme Alps study [42]. In another chamber study, approximately 6.4 mmol nitrate daily for 5 days (a very similar dose to our intervention) increased plasma nitrate levels by over 180 μM, yet this exacerbated AMS symptoms, worsened sense of effort and increased blood pressure during walking exercise in participants susceptible to AMS exposed to up to 6 h of acute hypoxia [43]. Meanwhile, in other field studies exposing subjects to hypobaric hypoxia, daily supplementation with approximately 10 mmoles nitrate demonstrated no improvement in sleep disordered breathing in lowlanders at altitudes between 3700 m and 4900 m in the Himalayas over 2 consecutive days [44], nor any improvement in adolescents’ AMS scores over 7 days at altitudes up to 5300 m [45].

Decreases in microcirculatory blood flow at higher altitudes in native lowlanders, similar to the findings reported here, have been well documented previously [16,17,24]. The findings demonstrated here do support these results again even though treatment with nitrate had no effect and all decreases seen were small – possibly due to the shorter duration of altitude exposure in this study compared to previous studies in the Himalaya [16,17,24]. Notably though, reduced microcirculatory blood flow was previously found to be associated with higher plasma nitrite concentrations [24]. The fact that in the Xtreme Alps study, plasma nitrite concentrations decreased at altitude compared to sea level - despite dietary nitrate supplementation - is a curious finding and may explain the lack of physiological acclimatisation responses seen in this cohort.

We cannot exclude that an even higher nitrate dose might have affected microvascular function. We are aware that more recent studies have used higher doses of nitrate and also investigated the dose-response curve of nitrate [46]; however none of these findings were available when our study was originally designed (2008/09). We have demonstrated previously that the dietary nitrate contained in this beetroot supplement was subject to enterosalivary circulation with consecutive reduction of nitrate to nitrite in the oral cavity, resulting in highly significant increases in nitrite levels in saliva and significant increases of NO in exhaled breath as well as exhaled breath condensate [30]. Why this increased availability in oral nitrite did not also translate into increased concentrations of nitrite in blood plasma remains unclear.

One possible explanation might be that the pharmacological effects of nitrate are critically dependent on the dosing regimen and thus not simply a function of the total dose administered and steady state-concentrations achieved. Unlike in other previous studies, the nitrate supplementation in our Xtreme Alps study was administered in three equally divided doses throughout the day with the aim of maintaining more consistent elevations in plasma concentrations of nitrate and related metabolic products [30]. This may have translated into lower peak concentrations of nitrite, nitroso and nitrosyl products and interconnected elements of the Reactive Species Interactome, consequently reducing the magnitude of the system's response [47]. In support of this suggestion, other studies administering high dose nitrate in divided portions over a more sustained period of time (4 week intervals) have also failed to demonstrate measurable decreases in blood pressure normally seen in response to an acute nitrate administration [48]. Alternatively, as the authors of this particular study discuss, the effects of sustained increases in plasma nitrate may chronically alter enzyme expression, and prolonged exposure to other dietary factors (such as sulfate) may further modify nitrate's effects [49,50]. Moreover, there now appears to be less evidence to support the original concept of hypoxia-dependent nitrite/NO-mediated increases in blood flow following nitrate supplementation. Indeed, most recent investigations in mice have demonstrated that bolus administration of nitrite induces microvascular dilatation in an NO-independent manner, with blood pressure changes secondary to redox-induced dimerization of protein kinase G1α, independent of either NO or cGMP [51]. This effect may also underlie the concerted cardio-protective response to brief elevations in plasma nitrite described previously, which were associated with a number of redox, metabolic and cardiac signalling changes [52]. Whether these results are translatable to humans remains to be investigated.

Our observations might also suggest that nitrite utilisation is enhanced in hypobaric hypoxia and/or that nitrite is being sequestered in other body compartments that have not been measured here. Equally little is known currently about the elimination and renal handling of nitrite and nitrate, although a recent study suggested that either one or both of these species may have direct effects on the renal tubular bed [53]. Given that altitude exposure alone (without any supplementation) affects renal function [54], the combined effect of taking nitrate supplementation at altitude on renal function warrants further investigation. Similarly, so does the effect beetroot-based juices have on nitrite levels in other body compartments, as well as on whole body exercise performance. Circulating concentrations of nitrite and nitrate in blood will ultimately be determined by the balance of dietary intake; endogenous NO production and its conversion to nitrate, enterosalivary circulation and microbial reduction to nitrite; as well as the excretion and uptake (but possibly also the release) of nitrite and nitrate by tissues. Relatively little is known about these processes in humans.

### 4.1 Strengths and limitations

To our knowledge, the Xtreme Alps study remains one of the largest studies to date investigating the effects of dietary nitrate supplementation under hypobaric hypoxia performed under field conditions at high altitude. Using intervention and placebo supplements (identical both in appearance and taste) allowed the study to use a double-blinded design and added reliability despite the many challenges that performing a study of this nature carries. Space on the mountain limited overall sample sizes and therefore the power of the study; potentially both increasing the risk of type 2 errors, and also reducing the positive predictive value of statistically significant results (the so-called ‘winner's curse’) [55]. We attempted to overcome this by taking two separate trek groups and planning identical (staggered) ascent profiles to allow direct comparison, however an incoming storm forced one trek to ascend to 4559 m one day earlier than the other. This has been accounted for, as much as possible, in the statistical model used for data analysis, and trends were still similar across both trekking groups. As each subject started their intervention juice drink three days before each testing period (both at sea level and at altitude) we were unable to measure untreated baseline values, and equally we could not include a full ‘intervention-free’ control group without further compromising the size of each intervention group. However, the few measurements that were taken on untreated members of laboratory staff all closely matched values of the placebo group, as we have reported previously [30]. Even though our cohort was predominately comprised of young males with some previous altitude experience this is actually a representative sample of the population who visit high altitude regions considering that most patients seen at the Everest Base Camp clinic over the last ten years have been males below 40 years of age [56].

## 5 Conclusions

Dietary nitrate supplementation at altitude can significantly increase levels of circulating nitrate and other NO-related metabolites. However, despite these marked biochemical changes, we did not identify any significant alteration to sublingual or forearm microcirculatory flow whilst living at 4559 m for over five days. This is consistent with the lack of effects on blood pressure, respiratory function and peripheral oxygen saturation reported by us previously in response to treatment with dietary nitrate supplementation [30]. However, this lack of improvement to small vessel function at altitude does not necessarily mean that dietary nitrate supplementation will have no effect on other organ systems or confers no benefit on overall exercise performance at altitude, and both of these areas merit further investigation.

## Funding

Xtreme Alps received charitable support from the Friends of University College Hospital NHS Foundation Trust as well as unrestricted research funding from Smiths Medical Ltd. and Deltex Medical Ltd. MP, LH and MF acknowledge support from the Faculty of Medicine, University of Southampton. Part of the work carried out at the University of Warwick was supported by funds from the Medical Research Council (Strategic Appointment Scheme, to MF). None of the funding bodies or the institutions the authors are affiliated with had any role in the study design; collection, analysis and interpretation of data; manuscript preparation, or decision to publish. AC is funded by a Southampton NIHR BRC Fellowship.

## Declaration of competing interest

MPWG serves on the medical advisory board of Sphere Medical Ltd and is a director of Oxygen Control Systems Ltd. He has received honoraria for speaking for and/or travel expenses from BOC Medical (Linde Group), Edwards Lifesciences and Cortex GmBH. MPWG leads the Xtreme- Everest Oxygen Research Consortium and the Fit-4-Surgery research collaboration. MPWG serves as the UK NIHR CRN national specialty group lead for Anaesthesia Perioperative Medicine and Pain and is an elected council member of the Royal College of Anaesthetists, and President of the Critical Care Medicine Section of the Royal Society of Medicine. DM has received lecture and consultancy fees from Siemens Healthineers and Edwards LifeSciences. MGM is a paid Consultant for Edwards Lifesciences; his University Chair is sponsored by Smiths Medical; he is the founding Editor of the Journal Perioperative Medicine and sits on the Editorial Board of the British Journal of Anaesthesia; he is the Editor-in-Chief of TopMedTalk.
